# *catena*-Poly[[aquanickel(II)]-μ-pyridine-2,6-dicarboxylato-[aquanickel(II)]-μ-2,5-di-4-pyridyl-1,3,4-thiadiazole]

**DOI:** 10.1107/S1600536809012628

**Published:** 2009-04-08

**Authors:** Xin-Yan Zhang

**Affiliations:** a Li Shui Vocational & Technical College, Lishui, Zhejiang 323000, People’s Republic of China

## Abstract

The two independent Ni^II^ ions in the one-dimensional title complex, [Ni_2_(C_7_H_3_NO_4_)_2_(C_12_H_8_N_4_S)(H_2_O)_2_]_*n*_ or [Ni_2_(pydc)_2_(bpt)(H_2_O)_2_]_*n*_ (H_2_pydc = pyridine-2,6-dicarboxylic acid and bpt = 2,5-di-4-pyrid­yl-1,3,4-thia­diazole), have different coordination environments. One Ni^II^ ion is in a slightly-distorted octa­hedral coordination environment formed by three O atoms from two adjacent pydc ligands, two N atoms from bpt and pydc ligands, and one water mol­ecule, while the other Ni^II^ ion is in distorted square-pyramidal geometry, coordinated by two O atoms from two carboxyl­ate groups and two N atoms from the pyridine rings of the pydc and bpt ligands in the basal plane, while a coordinated water mol­ecule occupies the apical site. In the crystal structure, the H atoms of both water mol­ecules are involved in inter­molecular hydrogen bonds with the O atoms of uncoordinated carboxyl­ate groups, which link chains into a three-dimensional network.

## Related literature

For information on the types of ligands used for metal-organic frameworks, see: Zhang *et al.* (2005 ▶); Wen *et al.* (2007 ▶); Dong *et al.* (2003 ▶). 
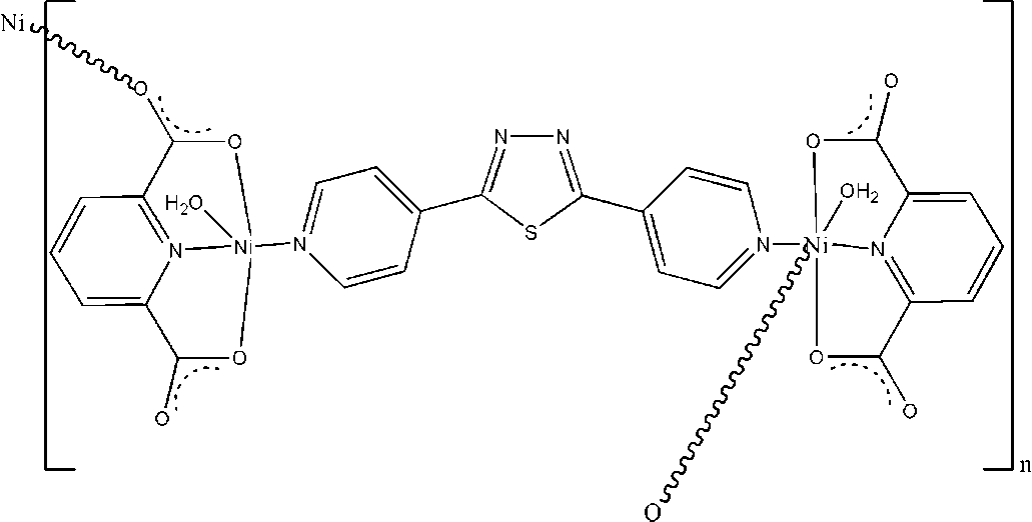


## Experimental

### 

#### Crystal data

[Ni_2_(C_7_H_3_NO_4_)_2_(C_12_H_8_N_4_S)(H_2_O)_2_]
*M* *_r_* = 723.94
Triclinic, 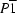

*a* = 8.2998 (12) Å
*b* = 10.0819 (15) Å
*c* = 17.318 (3) Å
α = 96.652 (2)°
β = 100.629 (2)°
γ = 108.077 (2)°
*V* = 1330.6 (3) Å^3^
*Z* = 2
Mo *K*α radiation
μ = 1.57 mm^−1^
*T* = 298 K
0.28 × 0.21 × 0.15 mm

#### Data collection

Bruker APEXII diffractometer
Absorption correction: multi-scan (*SADABS*; Bruker, 2004 ▶) *T*_min_ = 0.668, *T*_max_ = 0.799
9932 measured reflections
4735 independent reflections
3249 reflections with *I* > 2σ(*I*)
*R*_int_ = 0.038

#### Refinement

*R*[*F* ^2^ > 2σ(*F* ^2^)] = 0.046
*wR*(*F* ^2^) = 0.147
*S* = 0.97
4735 reflections
406 parameters
H-atom parameters constrained
Δρ_max_ = 0.43 e Å^−3^
Δρ_min_ = −0.54 e Å^−3^

### 

Data collection: *APEX2* (Bruker, 2004 ▶); cell refinement: *SMART* (Bruker, 2004 ▶); data reduction: *SAINT*; program(s) used to solve structure: *SHELXS97 *(Sheldrick, 2008 ▶); program(s) used to refine structure: *SHELXL97* (Sheldrick, 2008 ▶); molecular graphics: *ORTEPIII* (Burnett & Johnson, 1996 ▶) and *ORTEP-3 for Windows* (Farrugia, 1997 ▶); software used to prepare material for publication: *SHELXL97*.

## Supplementary Material

Crystal structure: contains datablocks I, global. DOI: 10.1107/S1600536809012628/lh2797sup1.cif

Structure factors: contains datablocks I. DOI: 10.1107/S1600536809012628/lh2797Isup2.hkl

Additional supplementary materials:  crystallographic information; 3D view; checkCIF report

## Figures and Tables

**Table 1:** Hydrogen-bond geometry (Å, °)

*D*—H⋯*A*	*D*—H	H⋯*A*	*D*⋯*A*	*D*—H⋯*A*
O9—H1*W*⋯O4^i^	0.83	1.99	2.815 (5)	178
O9—H2*W*⋯O8^ii^	0.83	1.95	2.755 (5)	161
O9—H2*W*⋯O7^ii^	0.83	2.43	3.093 (5)	138
O10—H3*W*⋯S1^iii^	0.85	2.75	3.601 (5)	179
O10—H4*W*⋯O5^iv^	0.85	1.97	2.814 (6)	168

Symmetry codes: (i) 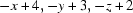; (ii) 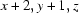; (iii) 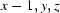; (iv) 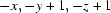.

**Fig. 1.**
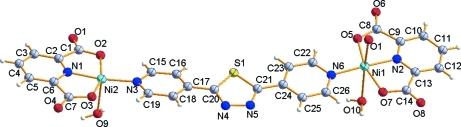
The asymmetric unit of the title compound showing the atom-labeling scheme. Displacement ellipsoids are shown at the 30% probability level. The symmetry related O1(-x-2, -1-y, -z) atom bonded to Ni1 is drawn to illustrate the slightly-distorted octahedral coordination environment.

## supplementary crystallographic information

### Comment

Recently, metal-organic frameworks have been obtained by using linear 4,4'-bipyridine, 1,2-bis(4-pyridyl)ethene and other bipyridine-like N,N'-donor ligands. However, the V-shaped N,N'-ligands, such as 2,5-di-4-pyridyl-1,3,4-oxadiazole, 4-amino-2,5-di-4-pyridyl-1,2,4-triazole, and 2,5-bis-(4- pyridyl)-1,3,4-thiadiazole find limited use as building blocks (Zhang *et al.*,  2005, Dong *et al.*,  2003). A study on the effect of angular N-containing ligands on the construction of coordination polymers in the presence of pyridine-2,6-dicarboxylic acid (pydc) is still not available (Wen *et al.*, 2007). In this paper, we report crystal structure of the title complex (I) containing an angular co-ligand.

The asymmetric unit of the title compound contains of two independent Ni^II^ ions, one bpt ligand, two pydc moieties, and two coordinated water molecules (Scheme 1, Figure 1). The two unique Ni^II^ ions have different coordination environments. Atom Ni1 adopts a slightly-distorted octahedral coordination formed by three O atoms from two adjacent pydc ligands, two N atoms from bpt and pydc ligands, and one coordinated water molecule, while atom Ni2 has distorted square-pyramidal coordination geometry and is coordinated by two oxygen atoms from two carboxylate groups and two N atoms from the pyridine rings of pydc and bpt, and one coordinated water molecule is located at the apical site. Both carboxylic groups are out of the plane of corresponding pyridine rings, with the dihedral angles 88.3 (5)° and 90.5 (7)°, respectively. The bpt ligands connect Ni atoms via two terminal pyridyl N atoms to form a binuclear unit. Furthermore, adjacent dimeric units are linked by carboxylic oxygen atoms of pydc ligands to form 1-D zigzag polymeric chains along [110]. In the crystal structure, H atoms of both water molecules are involved in hydrogen bonds with O atoms of uncoordinated carboxylate groups which link extended chains to form an three-dimensional network (Table 1).

### Experimental

A mixture of bpt (0.031 g, 0.15 mmol), NiSO_4_ (0.029 g, 0.13 mmol), and H_2_pydc (0.38g, 0.26mmol) in NaOH (0.2mL, 0.5M) and CH_3_CN(20 mL) solution, was stirred vigourously for 3 hours, and then then filtered. The resulting liquid was kept at room temperature and one week later single crystals suitable for X-ray diffraction measurements were formed.

### Refinement

The H atoms attached to C atoms were fixed geometrically and treated as riding with C—H = 0.93 Å with U_iso_(H) = 1.2U_eq_(C). The H atoms of water molecule were located in difference Fourier maps and refined in 'as found' positions with U_iso_(H) = 1.5U_eq_(O).

### Crystal data

**Table:**

[Ni_2_(C_7_H_3_NO_4_)_2_(C_12_H_8_N_4_S)(H_2_O)_2_]	*Z* = 2
*M**_r_* = 723.94	*F*(000) = 736
Triclinic, *P*1	*D*_x_ = 1.807 Mg m^−^^3^
Hall symbol: -P 1	Mo *K*α radiation, λ = 0.71073 Å
*a* = 8.2998 (12) Å	Cell parameters from 4735 reflections
*b* = 10.0819 (15) Å	θ = 2.3–25.2°
*c* = 17.318 (3) Å	µ = 1.57 mm^−^^1^
α = 96.652 (2)°	*T* = 298 K
β = 100.629 (2)°	Block, green
γ = 108.077 (2)°	0.28 × 0.21 × 0.15 mm
*V* = 1330.6 (3) Å^3^	

### Data collection

**Table:**

Bruker APEXII diffractometer	4735 independent reflections
Radiation source: fine-focus sealed tube	3249 reflections with *I* > 2σ(*I*)
graphite	*R*_int_ = 0.038
φ and ω scans	θ_max_ = 25.2°, θ_min_ = 2.3°
Absorption correction: multi-scan (*SADABS*; Bruker, 2004)	*h* = −9→9
*T*_min_ = 0.668, *T*_max_ = 0.799	*k* = −12→12
9932 measured reflections	*l* = −20→20

### Refinement

**Table:**

Refinement on *F*^2^	Primary atom site location: structure-invariant direct methods
Least-squares matrix: full	Secondary atom site location: difference Fourier map
*R*[*F*^2^ > 2σ(*F*^2^)] = 0.046	Hydrogen site location: inferred from neighbouring sites
*wR*(*F*^2^) = 0.147	H-atom parameters constrained
*S* = 0.97	*w* = 1/[σ^2^(*F*_o_^2^) + (0.0901*P*)^2^] where *P* = (*F*_o_^2^ + 2*F*_c_^2^)/3
4735 reflections	(Δ/σ)_max_ = 0.001
406 parameters	Δρ_max_ = 0.43 e Å^−^^3^
0 restraints	Δρ_min_ = −0.54 e Å^−^^3^

### Special details

**Table:**

Geometry. All esds (except the esd in the dihedral angle between two l.s. planes) are estimated using the full covariance matrix. The cell esds are taken into account individually in the estimation of esds in distances, angles and torsion angles; correlations between esds in cell parameters are only used when they are defined by crystal symmetry. An approximate (isotropic) treatment of cell esds is used for estimating esds involving l.s. planes.
Refinement. Refinement of F^2^ against ALL reflections. The weighted R-factor wR and goodness of fit S are based on F^2^, conventional R-factors R are based on F, with F set to zero for negative F^2^. The threshold expression of F^2^ > 2sigma(F^2^) is used only for calculating R-factors(gt) etc. and is not relevant to the choice of reflections for refinement. R-factors based on F^2^ are statistically about twice as large as those based on F, and R- factors based on ALL data will be even larger.

### Fractional atomic coordinates and isotropic or equivalent isotropic displacement parameters (Å^2^)

**Table:**

	*x*	*y*	*z*	*U*_iso_*/*U*_eq_	
Ni2	1.69956 (7)	1.19003 (6)	0.87739 (3)	0.02962 (19)	
Ni1	−0.14341 (8)	0.36762 (7)	0.60834 (4)	0.0429 (2)	
S1	0.77693 (15)	0.77425 (14)	0.74915 (8)	0.0431 (4)	
O1	1.8982 (4)	1.1719 (4)	0.6830 (2)	0.0488 (9)	
O2	1.7107 (4)	1.1265 (4)	0.76286 (19)	0.0402 (8)	
O3	1.7748 (4)	1.2547 (4)	0.99695 (19)	0.0403 (8)	
O4	2.0135 (4)	1.3775 (4)	1.0943 (2)	0.0455 (9)	
O5	−0.0806 (5)	0.2964 (4)	0.5084 (2)	0.0594 (11)	
O6	−0.1988 (6)	0.1538 (5)	0.3893 (2)	0.0734 (13)	
O7	−0.2846 (5)	0.4040 (4)	0.6856 (2)	0.0475 (9)	
O8	−0.5522 (5)	0.3199 (4)	0.7064 (2)	0.0547 (10)	
O9	1.6629 (4)	1.4007 (4)	0.8577 (2)	0.0427 (8)	
H1W	1.7581	1.4660	0.8704	0.064*	
H2W	1.6180	1.3885	0.8091	0.064*	
O10	−0.1970 (7)	0.5901 (5)	0.5634 (3)	0.0955 (16)	
H3W	−0.2039	0.6337	0.6070	0.143*	
H4W	−0.1103	0.6361	0.5461	0.143*	
N1	1.9442 (5)	1.2626 (4)	0.8884 (2)	0.0321 (9)	
N2	−0.3695 (5)	0.2512 (4)	0.5473 (2)	0.0406 (10)	
N3	1.4516 (5)	1.0892 (4)	0.8666 (2)	0.0340 (9)	
N4	0.8221 (5)	0.9038 (5)	0.8903 (2)	0.0449 (11)	
N5	0.6470 (5)	0.8299 (5)	0.8649 (2)	0.0442 (11)	
N6	0.0871 (5)	0.4965 (4)	0.6684 (2)	0.0390 (10)	
C1	1.8619 (6)	1.1791 (5)	0.7489 (3)	0.0339 (11)	
C2	2.0073 (6)	1.2550 (5)	0.8231 (3)	0.0323 (11)	
C3	2.1824 (6)	1.3118 (5)	0.8283 (3)	0.0372 (12)	
H3	2.2270	1.3079	0.7830	0.045*	
C4	2.2911 (6)	1.3751 (6)	0.9028 (3)	0.0467 (14)	
H4	2.4109	1.4131	0.9081	0.056*	
C5	2.2227 (6)	1.3823 (5)	0.9695 (3)	0.0435 (13)	
H5	2.2954	1.4258	1.0197	0.052*	
C6	2.0453 (6)	1.3240 (5)	0.9606 (3)	0.0329 (11)	
C7	1.9391 (6)	1.3217 (5)	1.0237 (3)	0.0362 (11)	
C8	−0.2127 (8)	0.2105 (6)	0.4531 (3)	0.0529 (15)	
C9	−0.3855 (7)	0.1851 (6)	0.4741 (3)	0.0462 (13)	
C10	−0.5481 (7)	0.1018 (6)	0.4266 (3)	0.0535 (15)	
H10	−0.5611	0.0541	0.3753	0.064*	
C11	−0.6908 (8)	0.0937 (6)	0.4600 (4)	0.0584 (16)	
H11	−0.8022	0.0410	0.4296	0.070*	
C12	−0.6711 (7)	0.1626 (6)	0.5379 (3)	0.0535 (15)	
H12	−0.7667	0.1554	0.5601	0.064*	
C13	−0.5039 (7)	0.2415 (6)	0.5803 (3)	0.0427 (13)	
C14	−0.4484 (7)	0.3255 (6)	0.6637 (3)	0.0420 (13)	
C15	1.3472 (6)	1.0279 (5)	0.7932 (3)	0.0370 (12)	
H15	1.3966	1.0319	0.7491	0.044*	
C16	1.1708 (6)	0.9599 (5)	0.7813 (3)	0.0357 (11)	
H16	1.1027	0.9185	0.7301	0.043*	
C17	1.0954 (6)	0.9536 (5)	0.8465 (3)	0.0304 (10)	
C18	1.2028 (6)	1.0150 (5)	0.9222 (3)	0.0347 (11)	
H18	1.1568	1.0116	0.9672	0.042*	
C19	1.3778 (6)	1.0807 (5)	0.9293 (3)	0.0357 (11)	
H19	1.4487	1.1214	0.9801	0.043*	
C20	0.9077 (6)	0.8850 (5)	0.8354 (3)	0.0323 (11)	
C21	0.6026 (6)	0.7566 (5)	0.7930 (3)	0.0328 (11)	
C22	0.2237 (6)	0.5116 (6)	0.6344 (3)	0.0460 (13)	
H22	0.2044	0.4638	0.5825	0.055*	
C23	0.3899 (7)	0.5949 (6)	0.6736 (3)	0.0469 (14)	
H23	0.4808	0.6017	0.6482	0.056*	
C24	0.4242 (6)	0.6691 (5)	0.7506 (3)	0.0343 (11)	
C25	0.2834 (6)	0.6548 (5)	0.7860 (3)	0.0388 (12)	
H25	0.2995	0.7013	0.8379	0.047*	
C26	0.1188 (6)	0.5695 (5)	0.7419 (3)	0.0385 (12)	
H26	0.0248	0.5629	0.7651	0.046*	

### Atomic displacement parameters (Å^2^)

**Table:**

	*U*^11^	*U*^22^	*U*^33^	*U*^12^	*U*^13^	*U*^23^
Ni2	0.0176 (3)	0.0381 (4)	0.0277 (3)	0.0029 (3)	0.0071 (2)	0.0001 (3)
Ni1	0.0297 (4)	0.0543 (5)	0.0314 (4)	−0.0011 (3)	0.0087 (3)	−0.0033 (3)
S1	0.0250 (7)	0.0552 (8)	0.0370 (7)	0.0022 (6)	0.0088 (5)	−0.0093 (6)
O1	0.040 (2)	0.066 (3)	0.033 (2)	0.0106 (18)	0.0136 (17)	−0.0056 (18)
O2	0.0252 (18)	0.048 (2)	0.0379 (19)	0.0035 (16)	0.0056 (15)	−0.0007 (16)
O3	0.0267 (18)	0.053 (2)	0.0344 (19)	0.0057 (16)	0.0079 (15)	0.0024 (16)
O4	0.036 (2)	0.057 (2)	0.035 (2)	0.0085 (18)	0.0064 (16)	−0.0009 (17)
O5	0.047 (2)	0.077 (3)	0.041 (2)	0.005 (2)	0.0176 (19)	−0.001 (2)
O6	0.074 (3)	0.084 (3)	0.050 (3)	0.012 (2)	0.025 (2)	−0.011 (2)
O7	0.038 (2)	0.050 (2)	0.044 (2)	0.0007 (17)	0.0134 (17)	−0.0023 (17)
O8	0.040 (2)	0.076 (3)	0.049 (2)	0.017 (2)	0.0185 (19)	0.005 (2)
O9	0.0343 (19)	0.048 (2)	0.041 (2)	0.0102 (16)	0.0086 (16)	0.0013 (16)
O10	0.097 (4)	0.097 (4)	0.089 (4)	0.015 (3)	0.040 (3)	0.025 (3)
N1	0.026 (2)	0.037 (2)	0.032 (2)	0.0074 (17)	0.0110 (17)	0.0031 (18)
N2	0.037 (2)	0.043 (3)	0.035 (2)	0.006 (2)	0.0076 (19)	0.003 (2)
N3	0.027 (2)	0.038 (2)	0.033 (2)	0.0054 (18)	0.0097 (18)	0.0025 (18)
N4	0.028 (2)	0.055 (3)	0.038 (3)	−0.001 (2)	0.0104 (19)	−0.006 (2)
N5	0.022 (2)	0.055 (3)	0.044 (3)	−0.0002 (19)	0.0111 (19)	−0.006 (2)
N6	0.029 (2)	0.041 (2)	0.038 (2)	0.0025 (19)	0.0070 (19)	0.002 (2)
C1	0.024 (3)	0.041 (3)	0.033 (3)	0.010 (2)	0.006 (2)	−0.002 (2)
C2	0.029 (3)	0.033 (3)	0.033 (3)	0.009 (2)	0.010 (2)	0.001 (2)
C3	0.024 (3)	0.046 (3)	0.042 (3)	0.011 (2)	0.014 (2)	0.005 (2)
C4	0.020 (3)	0.056 (3)	0.054 (4)	0.003 (2)	0.011 (2)	−0.003 (3)
C5	0.030 (3)	0.047 (3)	0.043 (3)	0.004 (2)	0.006 (2)	−0.009 (2)
C6	0.026 (3)	0.037 (3)	0.033 (3)	0.009 (2)	0.008 (2)	0.002 (2)
C7	0.031 (3)	0.041 (3)	0.034 (3)	0.009 (2)	0.010 (2)	0.004 (2)
C8	0.060 (4)	0.052 (4)	0.035 (3)	0.007 (3)	0.012 (3)	−0.002 (3)
C9	0.051 (3)	0.040 (3)	0.041 (3)	0.007 (3)	0.011 (3)	0.005 (2)
C10	0.055 (4)	0.048 (3)	0.039 (3)	0.001 (3)	−0.001 (3)	0.000 (3)
C11	0.047 (4)	0.059 (4)	0.049 (4)	0.004 (3)	−0.005 (3)	−0.002 (3)
C12	0.035 (3)	0.058 (4)	0.055 (4)	0.004 (3)	0.005 (3)	0.001 (3)
C13	0.036 (3)	0.045 (3)	0.044 (3)	0.010 (2)	0.010 (2)	0.007 (2)
C14	0.039 (3)	0.047 (3)	0.035 (3)	0.012 (3)	0.005 (2)	0.003 (2)
C15	0.027 (3)	0.045 (3)	0.031 (3)	0.005 (2)	0.008 (2)	−0.002 (2)
C16	0.026 (3)	0.042 (3)	0.032 (3)	0.004 (2)	0.008 (2)	0.000 (2)
C17	0.027 (2)	0.029 (3)	0.029 (3)	0.003 (2)	0.007 (2)	0.002 (2)
C18	0.027 (3)	0.038 (3)	0.035 (3)	0.003 (2)	0.013 (2)	0.007 (2)
C19	0.029 (3)	0.047 (3)	0.026 (3)	0.008 (2)	0.003 (2)	0.002 (2)
C20	0.026 (3)	0.033 (3)	0.031 (3)	0.004 (2)	0.004 (2)	0.001 (2)
C21	0.023 (2)	0.036 (3)	0.038 (3)	0.005 (2)	0.013 (2)	0.007 (2)
C22	0.032 (3)	0.057 (4)	0.040 (3)	0.004 (3)	0.010 (2)	−0.002 (3)
C23	0.031 (3)	0.057 (3)	0.046 (3)	0.010 (3)	0.012 (2)	−0.002 (3)
C24	0.030 (3)	0.032 (3)	0.038 (3)	0.008 (2)	0.007 (2)	0.000 (2)
C25	0.030 (3)	0.040 (3)	0.040 (3)	0.005 (2)	0.011 (2)	−0.002 (2)
C26	0.026 (3)	0.046 (3)	0.040 (3)	0.006 (2)	0.010 (2)	0.005 (2)

### Geometric parameters (Å, °)

**Table:**

Ni2—N1	1.897 (4)	C1—C2	1.523 (6)
Ni2—N3	1.954 (4)	C2—C3	1.368 (6)
Ni2—O3	2.015 (3)	C3—C4	1.381 (7)
Ni2—O2	2.040 (3)	C3—H3	0.9300
Ni2—O9	2.291 (3)	C4—C5	1.382 (7)
Ni1—N2	1.906 (4)	C4—H4	0.9300
Ni1—N6	1.960 (4)	C5—C6	1.377 (6)
Ni1—O7	2.007 (3)	C5—H5	0.9300
Ni1—O5	2.015 (4)	C6—C7	1.523 (6)
Ni1—O10	2.594 (5)	C8—C9	1.497 (8)
Ni1—O1^i^	2.556 (4)	C9—C10	1.389 (7)
S1—C20	1.708 (5)	C10—C11	1.395 (8)
S1—C21	1.726 (4)	C10—H10	0.9300
O1—C1	1.234 (5)	C11—C12	1.401 (8)
O2—C1	1.281 (5)	C11—H11	0.9300
O3—C7	1.286 (6)	C12—C13	1.379 (7)
O4—C7	1.240 (6)	C12—H12	0.9300
O5—C8	1.304 (7)	C13—C14	1.498 (7)
O6—C8	1.226 (6)	C15—C16	1.374 (6)
O7—C14	1.300 (6)	C15—H15	0.9300
O8—C14	1.229 (6)	C16—C17	1.387 (6)
O9—H1W	0.8282	C16—H16	0.9300
O9—H2W	0.8325	C17—C18	1.390 (6)
O10—H3W	0.8496	C17—C20	1.460 (6)
O10—H4W	0.8529	C18—C19	1.372 (6)
N1—C6	1.328 (6)	C18—H18	0.9300
N1—C2	1.334 (6)	C19—H19	0.9300
N2—C9	1.325 (6)	C21—C24	1.466 (6)
N2—C13	1.326 (6)	C22—C23	1.368 (7)
N3—C19	1.339 (6)	C22—H22	0.9300
N3—C15	1.353 (6)	C23—C24	1.384 (7)
N4—C20	1.318 (6)	C23—H23	0.9300
N4—N5	1.370 (5)	C24—C25	1.394 (6)
N5—C21	1.297 (6)	C25—C26	1.387 (7)
N6—C26	1.330 (6)	C25—H25	0.9300
N6—C22	1.348 (6)	C26—H26	0.9300
			
N1—Ni2—N3	171.86 (16)	N1—C6—C5	119.3 (4)
N1—Ni2—O3	80.99 (14)	N1—C6—C7	111.7 (4)
N3—Ni2—O3	99.57 (15)	C5—C6—C7	128.9 (4)
N1—Ni2—O2	80.38 (14)	O4—C7—O3	125.9 (4)
N3—Ni2—O2	97.88 (14)	O4—C7—C6	119.9 (4)
O3—Ni2—O2	160.06 (14)	O3—C7—C6	114.1 (4)
N1—Ni2—O9	93.67 (14)	O6—C8—O5	123.7 (6)
N3—Ni2—O9	94.42 (14)	O6—C8—C9	122.1 (5)
O3—Ni2—O9	92.34 (13)	O5—C8—C9	114.2 (5)
O2—Ni2—O9	95.87 (13)	N2—C9—C10	120.9 (5)
N2—Ni1—N6	176.70 (18)	N2—C9—C8	112.0 (5)
N2—Ni1—O7	80.93 (16)	C10—C9—C8	127.1 (5)
N6—Ni1—O7	98.94 (16)	C9—C10—C11	116.4 (5)
N2—Ni1—O5	80.32 (17)	C9—C10—H10	121.8
N6—Ni1—O5	99.67 (16)	C11—C10—H10	121.8
O7—Ni1—O5	161.16 (16)	C10—C11—C12	121.9 (5)
N2—Ni1—O10	90.19 (17)	C10—C11—H11	119.0
N6—Ni1—O10	86.53 (17)	C12—C11—H11	119.0
O7—Ni1—O10	81.20 (16)	C13—C12—C11	117.0 (5)
O5—Ni1—O10	97.04 (17)	C13—C12—H12	121.5
N2—Ni1—O1^i^	93.23 (15)	C11—C12—H12	121.5
N6—Ni1—O1^i^	90.06 (15)	N2—C13—C12	120.5 (5)
O7—Ni1—O1^i^	86.78 (13)	N2—C13—C14	112.0 (5)
O5—Ni1—O1^i^	96.07 (14)	C12—C13—C14	127.5 (5)
C20—S1—C21	87.9 (2)	O8—C14—O7	123.9 (5)
C1—O2—Ni2	113.8 (3)	O8—C14—C13	121.6 (5)
C7—O3—Ni2	114.5 (3)	O7—C14—C13	114.6 (4)
C8—O5—Ni1	114.6 (4)	N3—C15—C16	122.4 (4)
C14—O7—Ni1	113.8 (3)	N3—C15—H15	118.8
Ni2—O9—H1W	110.3	C16—C15—H15	118.8
Ni2—O9—H2W	105.3	C15—C16—C17	119.4 (5)
H1W—O9—H2W	111.2	C15—C16—H16	120.3
Ni1—O10—H3W	101.0	C17—C16—H16	120.3
Ni1—O10—H4W	108.8	C18—C17—C16	118.2 (4)
H3W—O10—H4W	112.9	C18—C17—C20	121.3 (4)
C6—N1—C2	122.7 (4)	C16—C17—C20	120.5 (4)
C6—N1—Ni2	118.3 (3)	C19—C18—C17	119.0 (4)
C2—N1—Ni2	118.9 (3)	C19—C18—H18	120.5
C9—N2—C13	123.2 (5)	C17—C18—H18	120.5
C9—N2—Ni1	118.8 (4)	N3—C19—C18	123.2 (4)
C13—N2—Ni1	118.0 (4)	N3—C19—H19	118.4
C19—N3—C15	117.7 (4)	C18—C19—H19	118.4
C19—N3—Ni2	122.7 (3)	N4—C20—C17	123.4 (4)
C15—N3—Ni2	119.6 (3)	N4—C20—S1	113.4 (3)
C20—N4—N5	112.2 (4)	C17—C20—S1	123.2 (3)
C21—N5—N4	113.4 (4)	N5—C21—C24	125.1 (4)
C26—N6—C22	117.2 (4)	N5—C21—S1	113.0 (3)
C26—N6—Ni1	124.1 (3)	C24—C21—S1	121.9 (4)
C22—N6—Ni1	118.7 (4)	N6—C22—C23	122.3 (5)
O1—C1—O2	126.5 (4)	N6—C22—H22	118.9
O1—C1—C2	118.9 (4)	C23—C22—H22	118.9
O2—C1—C2	114.6 (4)	C22—C23—C24	120.8 (5)
N1—C2—C3	120.5 (4)	C22—C23—H23	119.6
N1—C2—C1	111.2 (4)	C24—C23—H23	119.6
C3—C2—C1	128.3 (4)	C23—C24—C25	117.3 (5)
C2—C3—C4	118.2 (4)	C23—C24—C21	120.8 (4)
C2—C3—H3	120.9	C25—C24—C21	121.9 (4)
C4—C3—H3	120.9	C26—C25—C24	118.3 (5)
C3—C4—C5	120.3 (5)	C26—C25—H25	120.9
C3—C4—H4	119.9	C24—C25—H25	120.9
C5—C4—H4	119.9	N6—C26—C25	124.1 (5)
C6—C5—C4	119.0 (5)	N6—C26—H26	118.0
C6—C5—H5	120.5	C25—C26—H26	118.0
C4—C5—H5	120.5		
			
N1—Ni2—O2—C1	−9.6 (3)	N1—C6—C7—O3	3.7 (6)
N3—Ni2—O2—C1	178.5 (3)	C5—C6—C7—O3	−178.0 (5)
O3—Ni2—O2—C1	−30.6 (6)	Ni1—O5—C8—O6	−178.0 (5)
O9—Ni2—O2—C1	83.2 (3)	Ni1—O5—C8—C9	1.6 (6)
N1—Ni2—O3—C7	5.5 (3)	C13—N2—C9—C10	1.0 (8)
N3—Ni2—O3—C7	177.3 (3)	Ni1—N2—C9—C10	−178.4 (4)
O2—Ni2—O3—C7	26.5 (6)	C13—N2—C9—C8	−178.0 (5)
O9—Ni2—O3—C7	−87.8 (3)	Ni1—N2—C9—C8	2.5 (6)
N2—Ni1—O5—C8	−0.2 (4)	O6—C8—C9—N2	176.9 (5)
N6—Ni1—O5—C8	−176.9 (4)	O5—C8—C9—N2	−2.6 (7)
O7—Ni1—O5—C8	−5.8 (8)	O6—C8—C9—C10	−2.0 (10)
O10—Ni1—O5—C8	−89.2 (4)	O5—C8—C9—C10	178.4 (5)
N2—Ni1—O7—C14	6.8 (3)	N2—C9—C10—C11	0.6 (8)
N6—Ni1—O7—C14	−176.5 (3)	C8—C9—C10—C11	179.5 (5)
O5—Ni1—O7—C14	12.4 (7)	C9—C10—C11—C12	−1.6 (9)
O10—Ni1—O7—C14	98.4 (4)	C10—C11—C12—C13	1.1 (9)
O3—Ni2—N1—C6	−3.3 (3)	C9—N2—C13—C12	−1.6 (8)
O2—Ni2—N1—C6	−176.2 (4)	Ni1—N2—C13—C12	177.9 (4)
O9—Ni2—N1—C6	88.4 (4)	C9—N2—C13—C14	179.3 (5)
O3—Ni2—N1—C2	179.3 (4)	Ni1—N2—C13—C14	−1.3 (6)
O2—Ni2—N1—C2	6.4 (3)	C11—C12—C13—N2	0.5 (8)
O9—Ni2—N1—C2	−88.9 (4)	C11—C12—C13—C14	179.5 (5)
O7—Ni1—N2—C9	176.8 (4)	Ni1—O7—C14—O8	171.5 (4)
O5—Ni1—N2—C9	−1.4 (4)	Ni1—O7—C14—C13	−9.2 (5)
O10—Ni1—N2—C9	95.7 (4)	N2—C13—C14—O8	−173.6 (5)
O7—Ni1—N2—C13	−2.7 (4)	C12—C13—C14—O8	7.3 (9)
O5—Ni1—N2—C13	179.1 (4)	N2—C13—C14—O7	7.1 (6)
O10—Ni1—N2—C13	−83.8 (4)	C12—C13—C14—O7	−172.0 (5)
O3—Ni2—N3—C19	1.1 (4)	C19—N3—C15—C16	0.7 (7)
O2—Ni2—N3—C19	171.4 (4)	Ni2—N3—C15—C16	−177.6 (4)
O9—Ni2—N3—C19	−92.0 (4)	N3—C15—C16—C17	0.3 (8)
O3—Ni2—N3—C15	179.3 (4)	C15—C16—C17—C18	−1.0 (7)
O2—Ni2—N3—C15	−10.4 (4)	C15—C16—C17—C20	178.5 (4)
O9—Ni2—N3—C15	86.2 (4)	C16—C17—C18—C19	0.9 (7)
C20—N4—N5—C21	0.5 (6)	C20—C17—C18—C19	−178.7 (4)
O7—Ni1—N6—C26	5.0 (4)	C15—N3—C19—C18	−0.9 (7)
O5—Ni1—N6—C26	−177.9 (4)	Ni2—N3—C19—C18	177.4 (4)
O10—Ni1—N6—C26	85.5 (4)	C17—C18—C19—N3	0.1 (7)
O7—Ni1—N6—C22	−176.0 (4)	N5—N4—C20—C17	178.9 (4)
O5—Ni1—N6—C22	1.1 (4)	N5—N4—C20—S1	−0.2 (5)
O10—Ni1—N6—C22	−95.5 (4)	C18—C17—C20—N4	16.3 (7)
Ni2—O2—C1—O1	−170.4 (4)	C16—C17—C20—N4	−163.3 (5)
Ni2—O2—C1—C2	10.6 (5)	C18—C17—C20—S1	−164.7 (4)
C6—N1—C2—C3	−0.1 (7)	C16—C17—C20—S1	15.8 (7)
Ni2—N1—C2—C3	177.1 (3)	C21—S1—C20—N4	−0.1 (4)
C6—N1—C2—C1	−179.9 (4)	C21—S1—C20—C17	−179.2 (4)
Ni2—N1—C2—C1	−2.7 (5)	N4—N5—C21—C24	−179.2 (4)
O1—C1—C2—N1	175.3 (4)	N4—N5—C21—S1	−0.6 (6)
O2—C1—C2—N1	−5.7 (6)	C20—S1—C21—N5	0.4 (4)
O1—C1—C2—C3	−4.5 (8)	C20—S1—C21—C24	179.1 (4)
O2—C1—C2—C3	174.5 (5)	C26—N6—C22—C23	1.8 (8)
N1—C2—C3—C4	0.8 (7)	Ni1—N6—C22—C23	−177.2 (4)
C1—C2—C3—C4	−179.4 (5)	N6—C22—C23—C24	−0.7 (9)
C2—C3—C4—C5	−1.1 (8)	C22—C23—C24—C25	0.3 (8)
C3—C4—C5—C6	0.7 (8)	C22—C23—C24—C21	179.1 (5)
C2—N1—C6—C5	−0.3 (7)	N5—C21—C24—C23	179.8 (5)
Ni2—N1—C6—C5	−177.5 (4)	S1—C21—C24—C23	1.3 (7)
C2—N1—C6—C7	178.2 (4)	N5—C21—C24—C25	−1.5 (8)
Ni2—N1—C6—C7	1.0 (5)	S1—C21—C24—C25	180.0 (4)
C4—C5—C6—N1	0.0 (8)	C23—C24—C25—C26	−0.9 (7)
C4—C5—C6—C7	−178.2 (5)	C21—C24—C25—C26	−179.7 (4)
Ni2—O3—C7—O4	176.1 (4)	C22—N6—C26—C25	−2.6 (7)
Ni2—O3—C7—C6	−6.4 (5)	Ni1—N6—C26—C25	176.4 (4)
N1—C6—C7—O4	−178.5 (4)	C24—C25—C26—N6	2.2 (7)
C5—C6—C7—O4	−0.2 (8)		

Symmetry codes: (i) *x*−2, *y*−1, *z*.

### Hydrogen-bond geometry (Å, °)

**Table:**

*D*—H···*A*	*D*—H	H···*A*	*D*···*A*	*D*—H···*A*
O9—H1W···O4^ii^	0.83	1.99	2.815 (5)	178
O9—H2W···O8^iii^	0.83	1.95	2.755 (5)	161
O9—H2W···O7^iii^	0.83	2.43	3.093 (5)	138
O10—H3W···S1^iv^	0.85	2.75	3.601 (5)	179
O10—H4W···O5^v^	0.85	1.97	2.814 (6)	168

Symmetry codes: (ii) −*x*+4, −*y*+3, −*z*+2; (iii) *x*+2, *y*+1, *z*; (iv) *x*−1, *y*, *z*; (v) −*x*, −*y*+1, −*z*+1.
